# Long non-coding RNA urothelial carcinoma associated 1 induces cell replication by inhibiting BRG1 in 5637 cells

**DOI:** 10.3892/or.2014.3309

**Published:** 2014-07-03

**Authors:** XIUJUAN WANG, YANBING GONG, BO JIN, CHENGLIN WU, JIANMING YANG, LE WANG, ZHENG ZHANG, ZEBIN MAO

**Affiliations:** 1 Department of Biochemistry and Molecular Biology, Health Science Center, Peking University, Beijing 100191, P.R. China; 2 Department of Urology, Peking University First Hospital, Institute of Urology, Peking University, National Urological Cancer Center, Beijing 100034, P.R. China

**Keywords:** lncRNA, BRG1, UCA1

## Abstract

Long non-coding RNA urothelial carcinoma associated 1 (UCA1) was first identified in bladder cancer tissues. High expression of UCA1 in bladder cancer has suggested it may serve as a potential diagnostic molecular marker for bladder cancer. Subsequent research in bladder cancer cell lines showed that UCA1 can promote cell proliferation, but the underlying mechanism remains unknown. In the present study, we identified BRG1 as a UCA1 binding partner using an *in vitro* RNA pull-down assay, and RNA-binding protein immunoprecipitation assay confirmed UCA1-BRG binding in bladder cancer cells *in vivo*. BRG1 is a chromatin remodeling factor with reported tumor suppressor activities that directly upregulates levels of the p21 cell cycle inhibitor by binding sequences in the p21 promoter. Depletion of UCA1 by RNAi resulted in upregulated p21 levels and inhibition of cell replication, while overexpressed UCA1 reduced p21 protein and promoted cell growth. Notably, UCA1 downregulation of p21 and induction of cell proliferation antagonized the function of BRG1. UCA1 highly expressed tissue samples are often with BRG1 high expression. Furthermore, we found that UCA1 impairs both binding of BRG1 to the p21 promoter and chromatin remodeling activity of BRG1. Collectively, these results demonstrate that UCA1 promotes bladder cancer cell proliferation by inhibiting BRG1.

## Introduction

Over the last decade, genome-wide transcriptome studies have shown that the mammalian genome is abundantly transcribed and that at least 80% of this transcription is exclusively associated with long non-coding RNAs (lncRNAs; >200 bp), which map to intronic and intergenic regions (1,2). Initially, lncRNAs were regarded as ‘transcriptional noise’, since they lack the capacity to code for proteins (3). To date, a growing body of evidence indicates that lncRNAs are important players in a wide range of biological processes, including chromatin remodeling, gene transcription, mRNA translation and protein function (4–6).

Previous studies have found that the mechanisms underlying gene regulation also involve non-coding RNAs, including lncRNAs (7–9). The lncRNA molecular functions in gene regulation have been classified into four archetypes (5): archetype I, as signals, lncRNA expression can faithfully reflect the combinatorial actions of transcription factors or signaling pathways to indicate gene regulation in space and time; archetype II, as decoys, lncRNAs can titrate transcription factors and other proteins away from chromatin or titrate the protein factors into nuclear subdomains; archetype III, as guides, lncRNAs can recruit chromatin-modifying enzymes to target genes; and archetype IV, as scaffolds, lncRNAs can bring together multiple proteins to form ribonucleoprotein complexes. However, the study of lncRNA is still in its infancy (10,11), as only a small portion of lncRNAs has been examined for its biological activities and the molecular mechanisms of action have been characterized for very few lncRNAs.

Urothelial carcinoma associated 1 (UCA1), a novel lncRNA, has been reported to play a potential role in the progression of bladder cancer and could serve as a biomarker for diagnosis of bladder cancer (12,13). Ectopic expression of UCA1 lncRNA in BLS-211, a bladder cancer cell line, significantly enhanced tumorigenicity and invasive potential of cells (14). Other studies found that UCA1 is associated with tumor-linked genes such as WNT6 (15), CYP1A1 (16) and AURKC (17). These findings suggested that UCA1 may regulate some aspects of the tumorigenic processes, particularly in urothelial carcinoma, but its specific function and mechanisms remain unknown.

The BRG1 protein is the functional ATPase subunit of the mammalian SWI/SNF family of ATP-dependent chromatin remodeling complexes. BRG1 has a widespread role in tumor suppression (18), cell differentiation (19) and cellular senescence (20). Previous studies have demonstrated that BRG1 generally functions as a tumor suppressor via various mechanisms, including upregulation of the cell cycle inhibitor p21, a direct target gene of BRG1 (20,21).

In the present study, we first examined the function of UCA1 in 5637 bladder cancer cells, which express high levels of UCA1. We found that UCA1 plays an oncogene-like role in this bladder cancer cell line, which is consistent with previous reports. Furthermore, we found UCA1 promotes 5637 cell proliferation by antagonizing the activities of BRG1, by reducing its binding to the p21 promoter and inhibiting its chromatin remodeling activity.

## Materials and methods

### Cell culture and tissue samples

Human bladder cancer 5637 and EJ cells were cultured in RPMI-1640, T24 cells were cultured in McCoy’s 5A, and 293T and HeLa cells were cultured in Dulbecco’s modified Eagle’s medium, all supplemented with 10% fetal bovine serum. All cells were maintained in a humidified atmosphere of 5% CO_2_ at 37°C. All tissue samples were obtained from a tissue bank at the Institute of Urology (Peking University, China). These tissue samples were operative samples of bladder cancers and were diagnosed by pathology. Every pair of samples included one bladder cancer tissue sample and one normal bladder tissue sample. The licensing committee approved the experiments undertaken and both ethical approval and informed consent were obtained. The ethical approval document no. is IRB00001052-13057.

### Vector constructs

To generate lentivirus vector constructs stably expressing small interfering RNA (siRNA) targeting BRG1 or UCA1, a short double strand DNA sequence was cloned into the pll3.7 lentivirus vector. The oligonucleotides were as follows: iBRG1 sense, 5′-TCATGCACCAGATGCAC AAGTTCAAGAGACTTGTGCATCTGGTGCATGTTTTT TC-3′ and iBRG1 antisense, 5′-TCGAGAAAAAACATGCAC CAGATGCACAAGTCTCTTGAACTTGTGCATCTGGTG CATGA-3′ (22); iUCA1 sense, 5′-TGGTAATGTATCATCGG CTTAGTTCAAGAGACTAAGCCGATGATACATTACCTT TTTTC-3′ and iUCA1 antisense, 5′-TCGAGAAAAAAGGTA ATGTATCATCGGCTTAGTCTCTTGAACTAAGCCGATG ATACATTACCA-3′; and control sense, 5′-TTTCTCCGAACG TGTCACGTTTCAAGAGAACGTGACACGTTCGGAGAA TTTTTTC-3′ and control antisense, 5′-TCGAGAAAAAATT CTCCGAACGTGTCACGTTCTCTTGAAACGTGACACG TTCGGAGAAA-3′. The resulting constructs were designated as pll3.7-iBRG1, pll3.7-iUCA1 and pll3.7-NC. All constructs were confirmed by sequencing.

The UCA1 expressing lentivirus vector pZsG-UCA1 was generated by inserting full-length UCA1 into the pZsG vector, which contains a puromycin resistant gene. We used the primers 5′-(*Not*I)-GCGGCCGCTGACATTCTTCTGGACA ATGAGTC-3′ and 5′-(*Bam*HI)-GGATCCGGCATATTAGCT TTAATGTAGGTG-3′ to obtain full-length UCA1 cDNA from 5637 cells. PCR amplification was performed using High-Fidelity DNA polymerase (NEB, Ipswich, MA, USA) with the following conditions: 98°C, 10 sec; 55°C, 30 sec; 72°C, 30 sec, repeat 32 times. We cloned BRG1 into the pcDNA3.1 using *Eco*RV restriction site. We used the primers 5′-AGCTCCCGTGAAGATGTCCAC-3′ and 5′-TCTGCTGCTACCCGTTACTGCT-3′ to obtain BRG1 from pBJ5-hBRG1. PCR amplification was performed using High-Fidelity DNA polymerase with the following conditions: 98°C, 10 sec; 61°C, 30 sec; 72°C, 90 sec; for 32 cycles. The pZsG-UCA1 and pcDNA3.1-BRG1 vectors were confirmed by sequencing.

### Lentivirus packaging and infection

Lentivirus was produced by transfecting the packaging plasmid psPAX2, envelope plasmid pMD2.G and the experiment or control lentiviral vector (pll3.7 or pZsG with respective inserts) into 293T cells. Cells were transfected using VigoFect (Vigorous, Beijing, China) according to the manufacturer’s protocol. Viruses in the culture medium were harvested at 36 and 60 h after transfection and centrifuged at 4°C at 83,000 × g for 2 h. Viruses were resuspended with 100 μl phosphate-buffered saline (PBS) and stored at −80°C. Cells were infected in the presence of 8 μg/ml polybrene. After 6 h, the medium was changed and 24-h media was changed with the appropriate selection conditions.

### MTT and colony formation assays

For [3-(4,5-dimethylthiazol-2-yl)-2,5-diphenyltetrazolium bromide (MTT)] assay, 5637 cells (10^3^/well) were seeded into a 96-well plate and incubated at 37°C. Growth of 5637 cells was measured following addition of 20 μl of MTT (5 mg/ml) into the culture medium. MTT reduction was determined using an automated microplate reader (Bio-Rad, Hercules, CA, USA). All the assays were performed in triplicate, and the data are presented as means ± SD.

For colony formation assay, cells (~10^3^ cells) were plated onto 35-mm petri dishes. Cells were cultured for 10–14 days. Colonies were stained using crystal violet and counted.

### RNA pull-down assay

To generate antisense and sense UCA1 RNA oligonucleotides, we first inserted UCA1 DNA oligonucleotides with an *Eco*RI site at the 5′ end, and a *Bam*HI site at the 3′ end into pcDNA3.1(+) and pcDNA3.1(−) plasmids, respectively, which harbor a T7 promoter. They were confirmed by sequencing. We linearized the vectors with *Eco*RI or *Bam*HI to obtain pcDNA3.1(+)-UCA1-*Eco*RI and pcDNA3.1(−)-UCA1-*Bam*HI. We incubated T7 RNA polymerase biotin RNA labeling mix (Roche, Basel, Switzerland) and pcDNA3.1(+)-UCA1-*Eco*RI (or pcDNA3.1(−)-UCA1-*Bam*HI) DNA template *in vitro* according to the manufacturer’s instructions and then purified these RNAs by phenol chloroform extraction. RNA pull-down assays were performed with HeLa cell lysate as previously described (23).

### Identification of BRG1 by mass spectrometry

Proteins precipitated by RNA pull-down assays were subjected to NuPAGE 4–12% Bis-Tris gel electrophoresis and examined by silver stain using the Pierce Silver Stain kit (24612; Thermo Fisher Scientific, Rockford, IL, USA) according to the manufacturer’s instructions. Specific bands only in the sense UCA lane (Fig. 2A) were excised and analyzed by mass spectrometry (GeneSci Biotech Company, Beijing, China).

### RNA-binding protein immunoprecipitation assay

RNA-binding protein immunoprecipitation assay was performed with the Magna RIP RNA-Binding Protein Immunoprecipitation kit (17–700; Merck KGaA, Darmstadt, Germany, ) according to the manufacturer’s instructions. UCA1 (primer sequences as above) was detected from the pulled down RNA by real-time PCR with the primers 5′-GCCCAAG GAACATCTCACCAATTT-3′ and 5′-TTGAGGGGTCAG ACTTTTGACAAGG-3′ using the ABI PRISM 7500 sequence detection system (Applied Biosystems, Rockford, IL, USA) according to the manufacturer’s instructions. The PCR conditions were: 95°C, 30 sec; 60°C, 30 sec, repeat 40 times.

### RNA extraction and PCR

Total RNA was extracted using TRIzol reagent (Invitrogen, Carlsbad, CA, USA) according to the manufacturer’s protocol. First-strand cDNA was synthesized using SuperScript™ III first-strand kits (Invitrogen) for RT-PCR. The *p21* mRNA was analyzed by PCR on cDNA with primers 5′-GAAGACCATGTGGACCTGTCA-3′ and 5′-GGCTTCCTCTTGGAGAAGATCA-3′. *GAPDH* was used as an internal control, 5′-ACGGATTTGGTCGTATTGGG-3′ and 5′-TGATTTTGGAGGGATCTCGC-3′. The *UCA1* RNA was analyzed by PCR with the primers, 5′-GCCCAAGGAAC ATCTCACCAATTT-3′ and 5′-TTGAGGGGTCAGACTTTT GACAAGG-3′. The *BRG1* RNA was analyzed by PCR with the primers: 5′-AGTGCTGCTGTTCTGCCAAAT-3′ and 5′-GGCTCGTTGAAGGTTTTCAG-3′.

### Western blot analysis

Cells were lysed in RIPA buffer (Applygen, Beijing, China) and total cell lysates were separated by SDS-PAGE, transferred to PVDF membranes (Merck Millipore, Darmstadt, Germany), immunoblotted with antibodies, and visualized using a ChemiDoc XRS+ Imaging System (Bio-Rad) or film. Antibodies used for immunoblotting were anti-β-actin antibody (PM053; MBL, Japan) (1:5,000), anti-human p21 (3733-1; Abcam Epitomics, Cambridge, UK) (1:2,000), anti-H3K9me3 (49–1008; Novex, Carlsbad, CA, USA) (1:1,000), anti-H3K4m3 (ab8580) (1:2,000) and anti-human BRG1 antibodies (ab4081) (both from Abcam) (1:2,000). Signals were detected using secondary antibody anti-rabbit IgG-HRP (7077; Cell Signaling, Beverly, MA, USA) (1:5,000).

### Chromatin immunoprecipitation (ChIP) assays

ChIP assays were performed with the ChIP kit (Pierce, Cambridge, UK) according to the manufacturer’s instructions. Briefly, 5637 cells were transfected with pll3.7-NC or pll3.7-iUCA1 viruses and selected with G418 for 5 days. The post-confluent cells were then washed in PBS and fixed with 1% formaldehyde for 10 min at 37°C. Cells were harvested, washed twice and homogenized by bead beating. Chromatin DNA was sheared using ultrasound to a size of 0.5–1 kb. ChIP was performed overnight at 4°C using the BRG1 antibody (ab4081; Abcam) or an isotype control IgG. After a 1 h incubation in the presence of salmon sperm DNA/protein A agarose beads, the immunoprecipitated DNA/protein complexes were then washed and eluted from the beads with 1% SDS and 0.1 M NaHCO_3_ solution. Protein/DNA cross-links were reversed by adding 5 M NaCl and protein K at 65°C for 4 h. DNA was purified and amplified by PCR with primers for detecting human p21 promoter sequences: forward primer, 5′-GGAAATGTGTCCAGCGCACCAAC-3′ and reverse primer, 5′-CAGCGCGGCCCTGATATACAACC-3′.

### ATP hydrolysis assays

The measurements of the ATPase activity of BRG1 in the presence of nucleosome particles (using Nucleosome Assembly kit E5350S; NEB, Ipswich, MA, USA) was carried out as previously described (24). Briefly, 100 ng of reconstituted nucleosomes were mixed with 1 μl of BRG1 and 1 μl Ci of [γ-^32^P] ATP in a final volume of 10 μl (10 mM HEPES, pH 7.8, 50 mM KCl, 5 mM DTT, 0.5 mM PMSF, 200 g/ml BSA, 5% glycerol, 3.5 mM MgCl_2_). Aliquots of 1 μl were obtained at the time points indicated, and the reaction was stopped with 10 μl of gel loading buffer containing 90% formamide, 0.2% SDS, 10 mM EDTA and dyes. ATP hydrolysis was analyzed on 15% denaturing polyacrylamide gels. Gels were dried and exposed with phosphoimager screens, and quantified using the ImageQuant software.

### Micrococcal nuclease (MNase) assays

Cells were permeabilized with 0.01% L-a-lysophosphatidylcholine in 150 mM sucrose, 80 mM KCl, 35 mM HEPES pH 7.4, 5 mM K_2_HPO_4_, 5 mM MgCl_2_ and 0.5 mM CaCl_2_ for 90 sec, followed by digestion for 60 sec with 2 U/ml micrococcal nuclease (NEB) in 20 mM sucrose, 50 mM Tris-HCl pH 7.5, 50 mM NaCl and 2 mM CaCl_2_ at room temperature for various durations. Digestion of the DNA was arrested by adding 50 mM EDTA. DNA was then purified by Tris-buffered phenol/chloroform/isoamyl alcohol extraction. DNA was precipitated using 0.3 M NaOAc (pH 6.5) and two volumes of ethanol on dry ice for 30 min, and then resuspended in Tris-EDTA (pH 8.0). DNA concentration was evaluated with a spectrophotometer. DNA separation was performed by agarose (0.8%) gel electrophoresis.

### Statistical analysis

Results are expressed as means ± SD. The statistical significance of differences in experimental data was analyzed using the two-sample Student’s t-test. P<0.05 was considered to indicate a statistically significant difference.

## Results

### UCA1 promotes proliferation of 5637 cells

**Figure 1:**
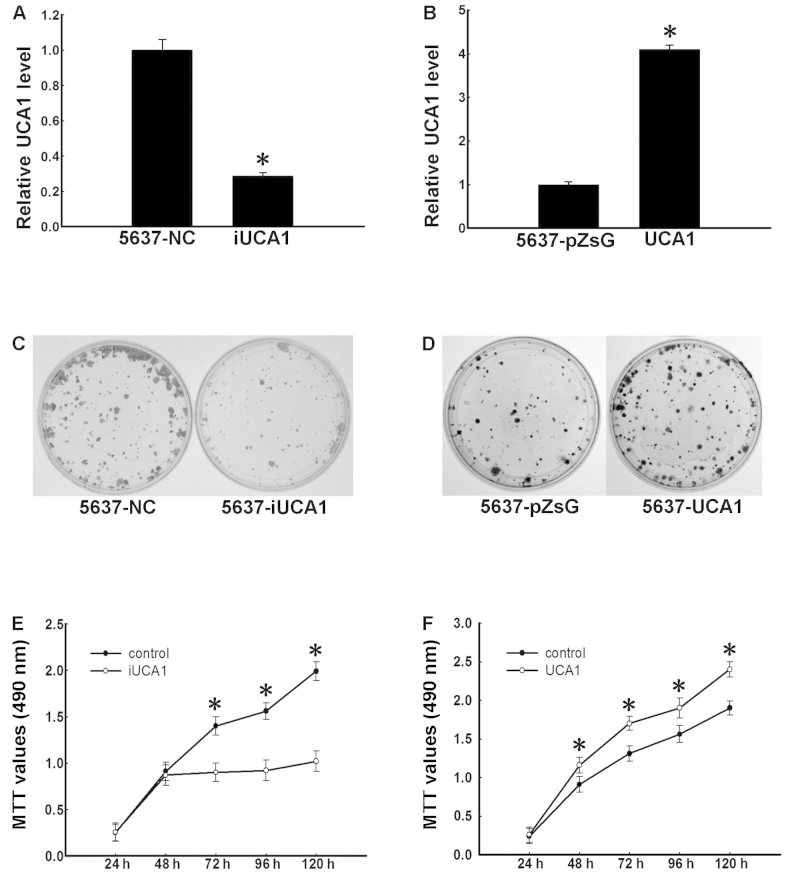
UCA1 promotes proliferation of 5637 cells. (A) Establishment of UCA1 knockdown 5637 cells. The 5637 cells were infected with pll3.7-NC (control) and pll3.7-iUCA1 viruses and selected with G418 for a week to generate 5637-NC and 5637-iUCA1 cells, respectively. Total RNA was isolated and the level of UCA1 was determined using real-time PCR. Data were normalized to *GAPDH* and expressed as the means ± SD of three independent experiments. ^*^P<0.05 (t-test). (B) Establishment of UCA1 overexpressing 5637 cells. The 5637 cells were infected with pZsG or pZsG-UCA1 viruses and selected with puromycin for 7 days to generate 5637-pZsG and 5637-UCA1 cells, respectively. Total RNA was isolated and the level of UCA1 was determined using real-time PCR. Data are expressed as the means ± SD of three independent experiments. ^*^P<0.05 (t-test). (C) Colony formation of 5637-NC and 5637-iUCA1 cells. The cells were cultured for 14 days. Colonies were stained using crystal violet. (D) Colony formation of 5637-pZsG and 5637-UCA1 cells. The cells were cultured for 14 days. Colonies were stained using crystal violet. (E) Growth curve of 5637-iUCA1 cells. Cellular proliferation was measured using MTT assays at 24, 48, 72, 96 and 120 h. The differences in data of 72, 96 and 120 h were significant, ^*^P<0.05 (t-test). (F) Growth curves of 5637-UCA1 cells. Cellular proliferation was measured using MTT assays at 24, 48, 72, 96 and 120 h. The differences in data of 48, 72, 96 and 120 h were significant, ^*^P<0.05 (t-test). UCA1, urothelial carcinoma associated 1.

**Figure 2:**
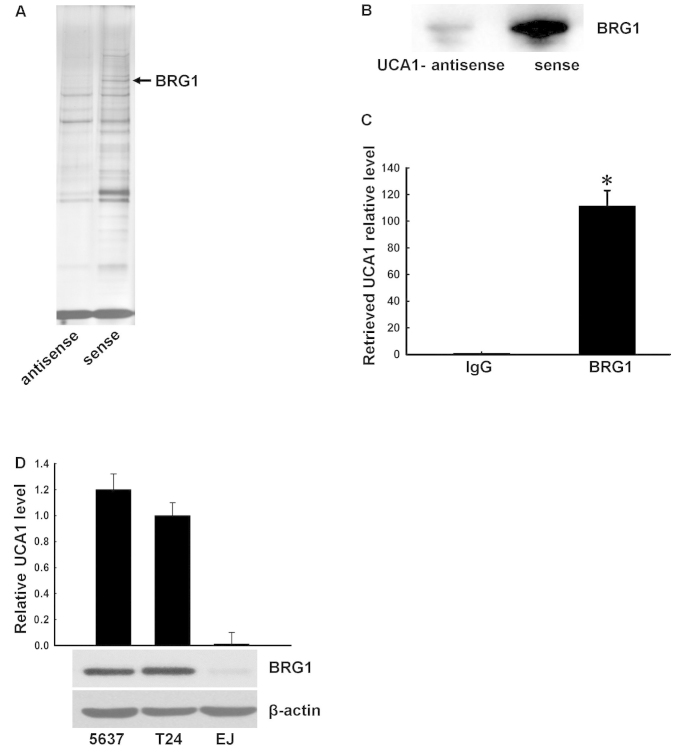
UCA1 binds to BRG1 *in vitro* and *in vivo*. (A) Electrophoresis of proteins bound to biotin-labeled sense and antisense UCA1 in NuPAGE 4–12% Bis-Tris gel. The biotin-labeled sense and antisense UCA1 were transcribed *in vitro* and incubated with HeLa cell lysate. The arrow shows the protein band for BRG1. (B) Western blot analysis of biotin-labeled sense and antisense UCA1-bound proteins using antibody against BRG1. (C) UCA1 binding BRG1 in 5637 cells was detected by RNA-binding protein immunoprecipitation assay. Antibody against BRG1 and mouse IgG (as a negative control) were used to pull-down RNAs in 5637 cells. The level of *UCA1* was determined using real-time PCR. Data were normalized to input and are expressed as the means ± SD of three independent experiments ^*^P<0.05 (t-test). (D) Expression profile analysis of three human bladder cancer cell lines. (Top) Total RNA was isolated and the level of *UCA1* was determined using real-time PCR. *GAPDH* was used as an internal control. (Bottom) BRG1 was determined by western blotting. β-actin was used as an internal control. UCA1, urothelial carcinoma associated 1.

To investigate the function of UCA1 lncRNA in urinary bladder cancer, we used the 5637 urinary bladder cell line that highly expresses UCA1 (Fig. 2D). Based on previous studies indicating that UCA1 exhibits tumorigenic activities, we first examined whether UCA1 promotes proliferation of 5637 cells. Cells were infected with lentiviruses harboring UCA1-shRNA or control-shRNA under G418 selection for >1 week, and knockdown of UCA1 in UCA1-shRNA-transfected cells was confirmed by real-time PCR (Fig. 1A). We next performed colony formation and MTT assays in the two cell lines. Colonies of UCA1-shRNA-transfected cells were fewer and smaller than those of the control group (Fig. 1C). Additionally, MTT assay results showed that UCA1-shRNA-infected cells grew slower than the control cells (Fig. 1E). To confirm these results, we generated a 5637 cell line ectopically expressing UCA1. Cells were infected with lentiviruses harboring UCA1 or controls under puromycin selection for 1 week, and UCA1 overexpression was confirmed by real-time PCR (Fig. 1B). Ectopic expression of UCA1 in 5637 cells promoted colony formation (Fig. 1D) and cell proliferation (Fig. 1F). These results indicate that UCA1 promotes 5637 cell proliferation, supporting its role as an oncogene-like factor.

### BRG1 is a target of UCA1

We next investigated the mechanism by which lncRNA UCA1 promotes cell proliferation by searching for possible protein factors that bind UCA1 using RNA pull-down assays. Biotin-labeled sense UCA1 RNA and antisense UCA1 RNA was transcribed *in vitro* and incubated with HeLa cell lysate. Avidin-labeled beads were added to precipitate biotin-labeled UCA1 RNA and any associated proteins. Samples were subjected to NuPAGE 4–12% Bis-Tris gel electrophoresis and examined by silver stain (Fig. 2A). Mass spectrometry of the band indicated by the arrow in the sense UCA1 lane in Fig. 2A revealed the band was likely BRG1, a chromatin remodeling factor. To verify UCA1-BRG binding, we next performed RNA pull-down and western blotting experiments in HeLa cells. As shown in Fig. 2B, western blotting confirmed UCA1 binding to BRG1 *in vitro*.

Next, we investigated UCA1 and BRG1 binding *in vivo* by RNA-binding protein immunoprecipitation (RIP) experiments. BRG1 antibody was used to immunoprecipitate BRG1 protein and associated RNAs in 5637 cells, and the presence of UCA1 RNA was determined by quantitative real-time PCR. Mouse IgG was used as control. The relative UCA1 RNA level in the BRG1 antibody immunoprecipitated samples was 111-fold higher compared with those in the IgG sample (Fig. 2C). Collectively, these data confirm that BRG1 binds UCA1 *in vitro* and *in vivo*. Notably, analysis of UCA1 and BRG1 expression in three bladder cancer cell lines (5637, T24 and EJ cells) revealed a relationship between BRG1 and UCA1 expression (Fig. 2D), suggesting that these two molecules may be functionally related.

### UCA1 is a suppressor of BRG1

**Figure 3:**
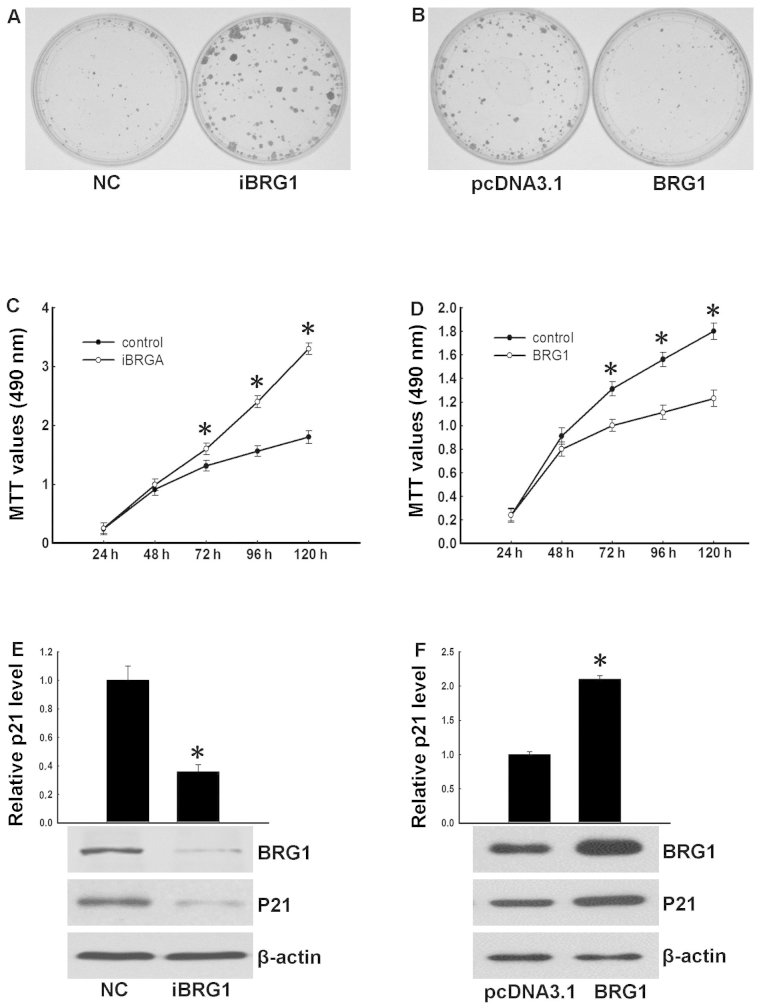
BRG1 plays a tumor suppressor role. (A) Colony formation of 5637-NC and 5637-iBRG1 cells. The cells were cultured for 14 days. Colonies were stained using crystal violet. (B) Colony formation of 5637-pcDNA3.1 and 5637-BRG1 cells. The cells were cultured for 14 days. Colonies were stained using crystal violet. (C) Growth curves of 5637 cells after transfection with BRG1 RNAi. Cellular proliferation was measured using MTT assay at 24, 48, 72, 96 and 120 h. The differences in data of 72, 96 and 120 h were significant, ^*^P<0.05 (t-test). (D) Growth curves of 5637 cells overexpressing BRG1. Cellular proliferation was measured using MTT assay at 24, 48, 72, 96 and 120 h. The differences in data of 72, 96 and 120 h were significant, ^*^P<0.05 (t-test). (E) The expression level of p21 in 5637-NC and 5637-iBRG1 cells was determined by real-time PCR. Data were normalized to *GAPDH* and are expressed as the means ± SD of three independent experiments, ^*^P<0.05 (t-test). Western blot analysis of BRG1 and P21 in 5637-NC and 5637-iBRG1 cells. β-actin served as the internal control. (F) The expression level of p21 in 5637-pcDNA3.1 and 5637-BRG1 cells was determined by real-time PCR. Data were normalized to *GAPDH* and are expressed as the means ± SD of three independent experiments, ^*^P<0.05 (t-test). Western blot analysis of BRG1 and p21 in 5637-pcDNA3.1 and 5637-BRG1 cells. β-actin served as the internal control.

To explore the significance of the association of UCA1 with BRG1, we first investigated BRG1 function in bladder cancer. To this end, BRG1 was depleted using a validated shRNA in 5637 cells. Western blot analysis confirmed that BRG1-shRNA could effectively suppress BRG1 expression (Fig. 3E). Consistent with previous reports, p21, a target gene of BRG1, displayed reduced expression at both the mRNA and protein levels in cells with BRG1 knockdown compared with controls. Furthermore, our results showed that BRG1 knockdown resulted in increased colony formation and cell growth (Fig. 3A and C). In contrast, overexpression of BRG1 in 5637 cells resulted in upregulated p21 expression (Fig. 3F) and suppressed colony formation and cell growth (Fig. 3B and D). These data suggest that BRG1 has anti-oncogenic functions in bladder cancer cells.

**Figure 4:**
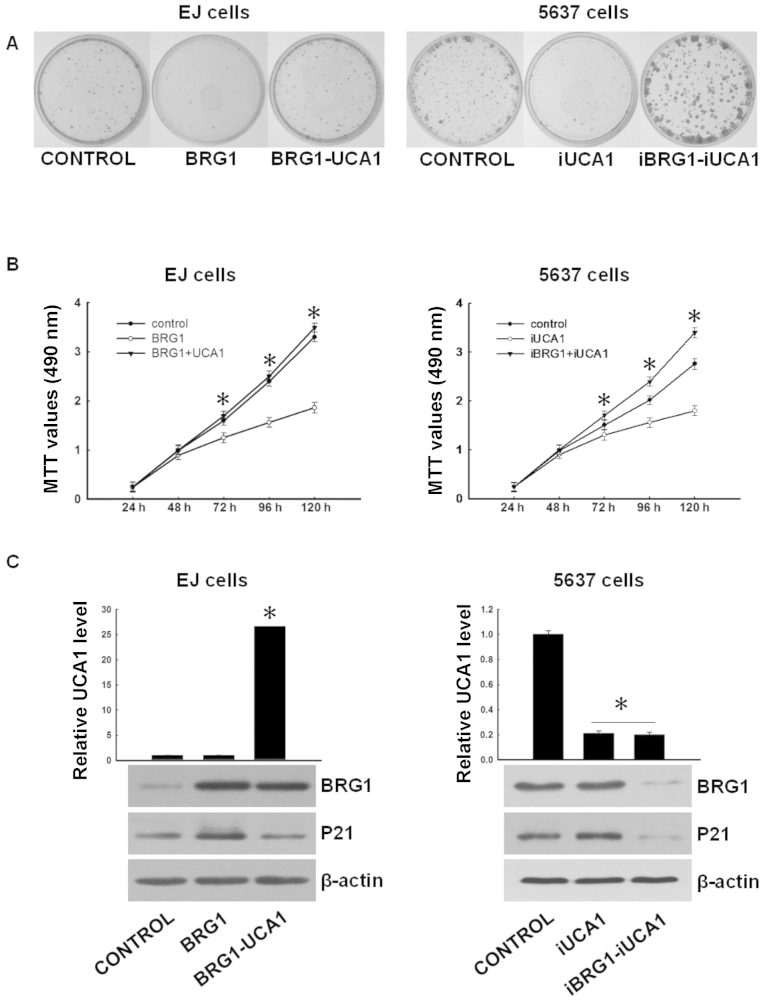
UCA1 antagonizes the tumor suppressing function of BRG1. (A) (Left) Colony formation of EJ control, EJ-BRG1 (overexpressed BRG1) and EJ-BRG1-UCA1 (overexpressed BRG1 and UCA1) cells. The cells were cultured with G418 for 10 days. Colonies were stained using crystal violet. (Right) Colony formation of 5637 control, 5637-iUCA1 (UCA1 knocked down) and 5637-iUCA1-iBRG1 (UCA1 and BRG1 both knocked down) cells. The cells were cultured with G418 and puromycin for 10 days. Colonies were stained using crystal violet. (B) (Left) Growth curves of EJ control, EJ-BRG1 and EJ-BRG1-UCA1 cells. Cellular proliferation was measured using MTT assays at 24, 48, 72, 96 and 120 h. The differences between EJ-BRG1 and EJ-BRG1-UCA1 were significant. ^*^P<0.05 (t-test). (Right) Growth curves of 5637 control, 5637-iUCA1 and 5637-iUCA1-iBRG1 cells. Cellular proliferation was measured using MTT assays at 24, 48, 72, 96 and 120 h. The differences between 5637-iUCA1 and 5637-iUCA1-iBRG1, 5637-iUCA1 and control were significant ^*^P<0.05 (t-test). (C) (Left) The expression levels of *UCA1* in EJ cells control EJ-BRG1 and EJ-BRG1-UCA1 were determined by real-time PCR. Data were normalized to *GAPDH* and are expressed as the means ± SD of three independent experiments, the *UCA1* level in EJ-BRG1-UCA1 was significantly high, ^*^P<0.05 (t-test). The expression levels of BRG1 and p21 were determined by western blotting with β-actin as the internal control. (Right) The expression levels of *UCA1* in 5637 cells control 5637-iUCA1 and 5637-iUCA1-iBRG1 were determined by real-time PCR. Data were normalized to *GAPDH* and are expressed as the means ± SD of three independent experiments. The effect of RNAi was significant ^*^P<0.05 (t-test). The expression levels of BRG1 and p21 were determined by western blotting with β-actin as the internal control. UCA1, urothelial carcinoma associated 1.

Since UCA1 interacts with BRG1 in 5637 cells, we hypothesized that UCA1 exerts proliferation-promoting functions by antagonizing BRG1. To confirm this, we performed several experiments. First, EJ cells, which lack BRG1 and UCA1 expression, were transfected with plasmids expressing BRG1, BRG1 and UCA1. Three days after drug selection, BRG1 and UCA1 expressions were confirmed by western blotting and real-time PCR (Fig. 4C, left). Colony formation and MTT assays showed that BRG1 overexpression suppressed colony formation and cell growth (Fig. 4A and B, left); however this effect was abrogated by overexpressing UCA1, suggesting that UCA1 antagonizes the growth suppressive function of BRG1.

To further analyze the functional association of BRG1 with UCA1, 5637 cells were transfected with UCA1-shRNA, UCA1-shRNA and BRG1-shRNA. Seven days after G418 selection, colony formation and MTT assay were performed. Results showed that UCA1-shRNA led to decreased colony formation and proliferation (Fig. 4A and B, right). However, BRG1-shRNA may weaken the suppressive role of UCA1-shRNA. Collectively, these data indicate that UCA1 acts as a suppressor of BRG1.

### UCA1 blocks recruitment of BRG1 to chromatin

**Figure 5:**
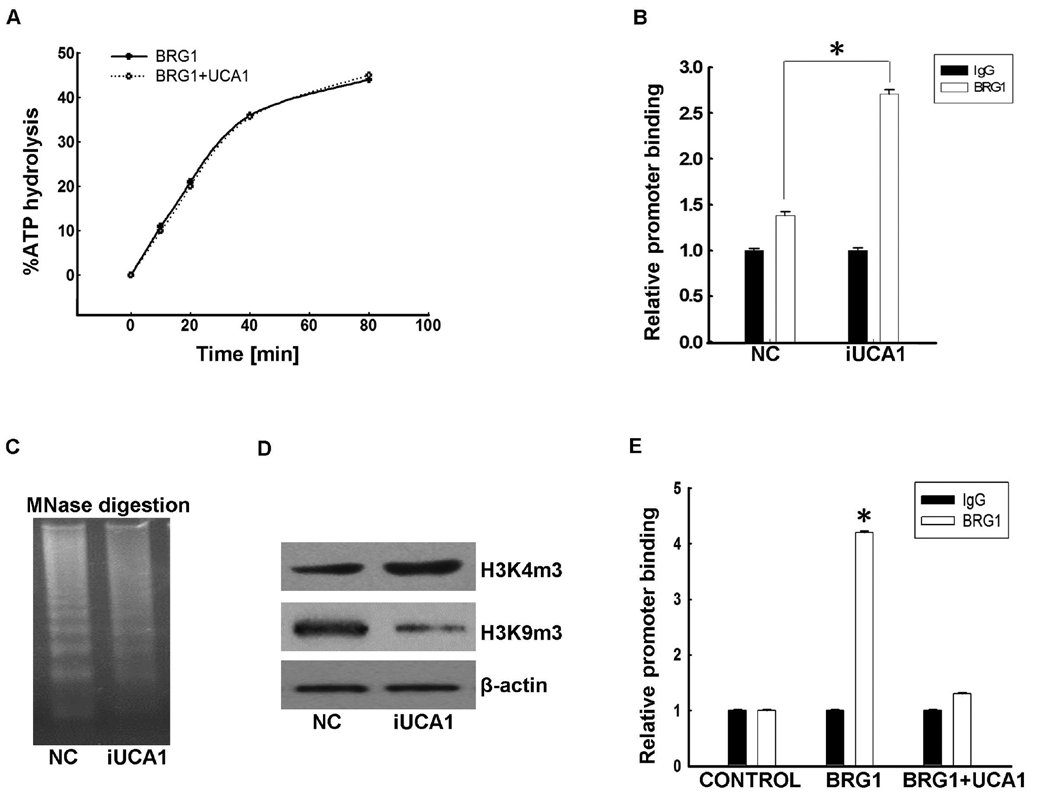
UCA1 blocks recruitment of BRG1 to chromatin. (A) UCA1 does not affect the ATPase activity of BRG1. The kinetics of BRG1-induced ATP hydrolysis were analyzed in the presence or absence of UCA1. (B) ChIP analysis of BRG1 binding to the p21 promoter in 5637-iUCA1. 5637-NC cells were used as the control. Genomic DNA was fixed and immunoprecipitated using anti-BRG1 antibody, with IgG as a negative control. Real-time PCR was performed using a primer set specific to the BRG1-binding site of p21 promoter. Data were normalized to input and are expressed as the means ± SD of three independent experiments. ^*^P<0.05 (t-test). (C) Micrococcal nuclease assay of 5637-NC, 5637-iUCA1. Same amounts of DNA were digested with micrococcal nuclease and electrophoresed. The image shows that nuclease digestion produced a laddering pattern. It is evident that DNA from 5637-iUCA1 is more sensitive to nuclease digestion. (D) Western blotting to detect histone proteins H3K4m3, H3K9m3 in 5637-NC, 5637-iUCA1. (E) ChIP analysis of BRG1 binding to the p21 promoter in EJ-BRG1, EJ-BRG1-UCA1. Genomic DNA was fixed and immunoprecipitated using anti-BRG1 antibody, with IgG as a negative control. Real-time PCR was performed using a primer set specific to the BRG1-binding site of p21 promoter. Data were normalized to input and are expressed as the means ± SD of three independent experiments. Overexpression of UCA1 in EJ-BRG1 led to decreased occupancy of p21 promoter by BRG1, ^*^P<0.05 (t-test). UCA1, urothelial carcinoma associated 1.

BRG1 is a catalytic subunit of the ATP-dependent nucleosome remodeling complex SWI/SNF and has helicase/ATPase activity. The SWI/SNF complex has no intrinsic ability to bind DNA and is commonly recruited to target promoters by other factors, such as transcription factors. We considered two possible pathways for UCA1 suppression of BRG1: i) UCA1 binds BRG1 and inhibits the ATPase activity of BRG1; ii) UCA1 binds BRG1 and blocks recruitment of BRG1 to target promoters. The *in vitro* ATPase activity assay results (Fig. 5A) showed that the presence of UCA1 did not result in changes of BRG1 ATPase activity, thus we suspected that UCA1 blocks recruitment of BRG1 to target promoters.

Indeed, ChIP analysis showed that depletion of UCA1 with shRNA enhanced BRG1 occupancy on the p21 promoter in 5637 cells (Fig. 5B). Furthermore, the genomic DNA in UCA1-shRNA cells became more sensitive to MNase digestion than that in cells with control-shRNA (Fig. 5C). Additionally, the level of H3K4m3, which is an active chromatin marker, were also increased in UCA1-shRNA cells, while H3K9m3, a repressive one, were decreased (Fig. 5D). Collectively, these data suggest that UCA1 suppresses the recruitment of BRG1 to its target promoters. To further confirm the above hypothesis, we ectopically expressed BRG1 and/or UCA1 in EJ cells as described above. ChIP results showed that BRG1 was enriched on the p21 promoter region in EJ cells overexpressing BRG1 alone (Fig. 5E). However, co-expression of UCA1 led to decreased occupancy of the p21 promoter by BRG1, and these suppressive effects of UCA1 showed dose-dependent characteristics.

### Expression of UCA1 is positively correlated with BRG1 in bladder cancer tissue specimens

**Figure 6:**
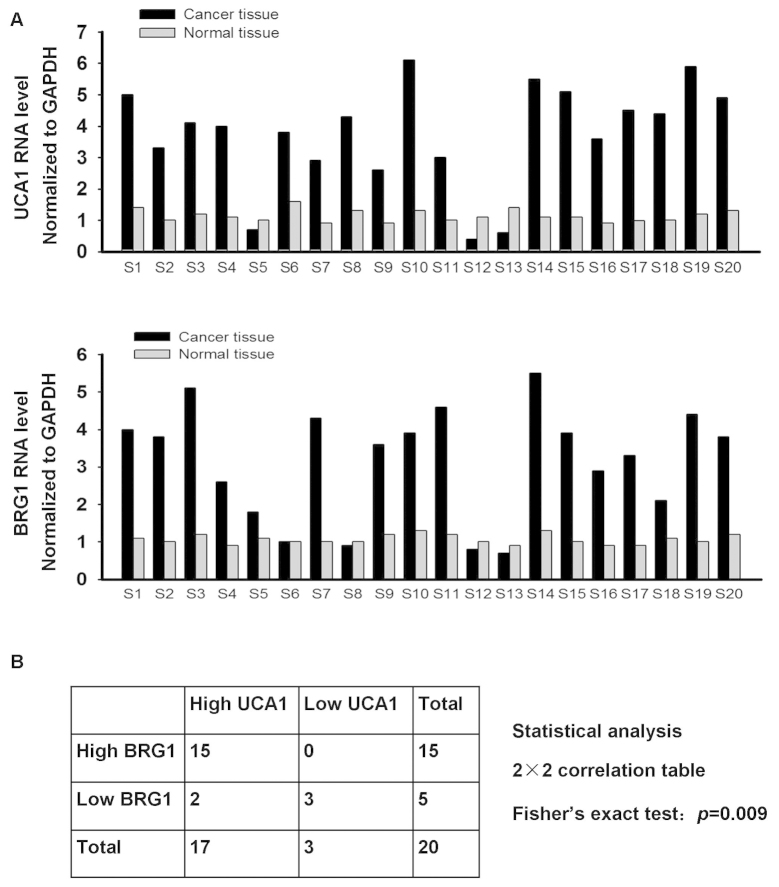
UCA1 expression correlates positively with BRG1 expression in bladder cancer tissue samples. (A) Real-time PCR was used to analyze UCA1 and BRG1 mRNA levels in the same samples. S, sample. (B) The status of UCA1 and BRG1 expression in bladder cancer specimens, >2-fold was identified as high expression. The 2×2 correlation table and Fisher’s exact test were used. The two-sided value P=0.009 was considered significant. UCA1, urothelial carcinoma associated 1.

The above observation that UCA1 promotion of proliferation is achieved at least partly by antagonizing BRG1 indicates that BRG1 is likely upregulated in bladder cancer. For this reason, we investigated BRG1 and UCA1 expression in tissue specimens of 20 bladder cancer patients with real-time PCR. As shown in Fig. 6A, 17 out of 20 bladder cancer tissue samples showed elevated UCA1 mRNA levels, whereas elevated BRG1 mRNA levels were detected in 15 cancer tissue samples. We used Fisher’s exact test to analyze the correlation between UCA1 and BRG1 (Fig. 6B). The results indicated a positive correlation between BRG1 expression and UCA1 expression and were significant, P=0.009. These data suggest that UCA1 antagonizes the suppressive effect of BRG1 on bladder cancer cells *in vivo*.

## Discussion

The long non-coding RNA UCA1 was first identified and termed by Wang *et al* in bladder cancer tissues. The authors found that UCA1 could serve as a potential biomarker for bladder cancer since it is specifically highly expressed in bladder cancer (12). Previous studies reported that UCA1 seems to function as a tumor-promoting factor in bladder cancers (14,25). However, the general function and underlying mechanisms of UCA1 in bladder cancer cells have remained elusive and require further investigation. In the present study, BRG1 was identified as target protein of UCA1. Our data showed that BRG1 exhibits anti-oncogenic features in bladder cancer cells. UCA1 bound BRG1 and antagonized its suppressive effects in bladder cancer cells. UCA1 blocked recruitment of BRG1 to its target promoter, and thus led to reduced expression of p21 and accelerated cell growth.

Three possibilities exist for the mechanisms underlying UCA1 blockage of BRG1 recruitment to promoter regions: i) in addition to BRG1, UCA1 binds another protein that blocks BRG1 access to promoter regions; ii) BRG1 is recruited to target promoter through specific transcription factors (26,27), thus UCA1 may hinder BRG1 interaction with specific transcription factors; and iii) UCA1 titrates BRG1 away from chromatin. Our studies cannot distinguish between these mechanisms. Titration would be a simple mechanism to prevent BRG1 access to promoter sequences even without impairing BRG1 ATPase activity. Furthermore, we cannot rule out the possibility of multiple and coexisting mechanisms of BRG1 control.

The present study revealed a notable phenomenon, namely, that although BRG1 has an antiproliferative role, its expression is elevated in bladder cancer tissue samples. Analysis of cDNA sequences showed that BRG1 mutation did not take place in these tissue samples (data not shown), thus, the possibility that mutation leads to inactivation of BRG1 function is ruled out. The elevated expression of BRG1 is likely associated with cellular senescence, which is an important tumor suppression mechanism. Upon exposure to external insults such as γ-irradiation or constitutively active oncogenes, normal cells become senescent. If senescence is inhibited, however, cells become prone to carcinogenesis. Previous studies have shown that overexpression of BRG1 may lead to cellular senescence (28). Additionally, BRG1 is required for formation of senescence-associated heterochromatin foci (SAHF) (20). We speculate that external insults lead to increased expression of BRG1, thus inducing cell senescence, and that UCA1 suppresses BRG1-mediated senescence, thus promoting cells carcinogenesis. P21 is also associated with cell senescence (29,30) and its increased level by BRG1 is consistent with levels in senescent cells. Our studies cannot confirm that p21 plays a key role in UCA1 inhibition of BRG1, since BRG1 has widespread functions via widespread pathways. However, p21 can be a clear indicator of BRG1 activity in 5637 cells.
